# Use of Machine Learning Models to Differentiate Neurodevelopment Conditions Through Digitally Collected Data: Cross-Sectional Questionnaire Study

**DOI:** 10.2196/54577

**Published:** 2024-07-29

**Authors:** Silvia Grazioli, Alessandro Crippa, Noemi Buo, Silvia Busti Ceccarelli, Massimo Molteni, Maria Nobile, Antonio Salandi, Sara Trabattoni, Gabriele Caselli, Paola Colombo

**Affiliations:** 1 Child Psychopathology Unit Scientific Institute IRCCS Eugenio Medea Bosisio Parini Italy; 2 Department of Psychology Sigmund Freud University Milan Italy; 3 Studi Cognitivi Cognitive Psychotherapy School and Research Centre Milan Italy

**Keywords:** digital-aided clinical assessment, machine learning, random forest, logistic regression, computational psychometrics, telemedicine, neurodevelopmental conditions, parent-report questionnaires, attention-deficit/hyperactivity disorder, autism spectrum disorder, ASD, autism, autistic, attention deficit, hyperactivity, classification

## Abstract

**Background:**

Diagnosis of child and adolescent psychopathologies involves a multifaceted approach, integrating clinical observations, behavioral assessments, medical history, cognitive testing, and familial context information. Digital technologies, especially internet-based platforms for administering caregiver-rated questionnaires, are increasingly used in this field, particularly during the screening phase. The ascent of digital platforms for data collection has propelled advanced psychopathology classification methods such as supervised machine learning (ML) into the forefront of both research and clinical environments. This shift, recently called psycho-informatics, has been facilitated by gradually incorporating computational devices into clinical workflows. However, an actual integration between telemedicine and the ML approach has yet to be fulfilled.

**Objective:**

Under these premises, exploring the potential of ML applications for analyzing digitally collected data may have significant implications for supporting the clinical practice of diagnosing early psychopathology. The purpose of this study was, therefore, to exploit ML models for the classification of attention-deficit/hyperactivity disorder (ADHD) and autism spectrum disorder (ASD) using internet-based parent-reported socio-anamnestic data, aiming at obtaining accurate predictive models for new help-seeking families.

**Methods:**

In this retrospective, single-center observational study, socio-anamnestic data were collected from 1688 children and adolescents referred for suspected neurodevelopmental conditions. The data included sociodemographic, clinical, environmental, and developmental factors, collected remotely through the first Italian internet-based screening tool for neurodevelopmental disorders, the Medea Information and Clinical Assessment On-Line (MedicalBIT). Random forest (RF), decision tree, and logistic regression models were developed and evaluated using classification accuracy, sensitivity, specificity, and importance of independent variables.

**Results:**

The RF model demonstrated robust accuracy, achieving 84% (95% CI 82-85; *P*<.001) for ADHD and 86% (95% CI 84-87; *P*<.001) for ASD classifications. Sensitivities were also high, with 93% for ADHD and 95% for ASD. In contrast, the DT and LR models exhibited lower accuracy (DT 74%, 95% CI 71-77; *P*<.001 for ADHD; DT 79%, 95% CI 77-82; *P*<.001 for ASD; LR 61%, 95% CI 57-64; *P*<.001 for ADHD; LR 63%, 95% CI 60-67; *P*<.001 for ASD) and sensitivities (DT: 82% for ADHD and 88% for ASD; LR: 62% for ADHD and 68% for ASD). The independent variables considered for classification differed in importance between the 2 models, reflecting the distinct characteristics of the 3 ML approaches.

**Conclusions:**

This study highlights the potential of ML models, particularly RF, in enhancing the diagnostic process of child and adolescent psychopathology. Altogether, the current findings underscore the significance of leveraging digital platforms and computational techniques in the diagnostic process. While interpretability remains crucial, the developed approach might provide valuable screening tools for clinicians, highlighting the significance of embedding computational techniques in the diagnostic process.

## Introduction

Child and adolescent psychopathology entails a complex diagnostic process that includes clinical observations, behavioral assessments, medical history evaluations, cognitive testing, and familial contexts. This comprehensive approach aligns with the recommendations outlined in the National Institute for Health and Care Excellence Guidelines [1], emphasizing the significance of a multifaceted understanding of these conditions.

In contemporary diagnostic practice, the adoption of digital technologies has gained remarkable prominence, particularly in the digital administration of caregiver-rated questionnaires focused on capturing neurodevelopmental and behavioral symptoms [2,3]. This shift toward digitalization (often called psycho-informatics) is fueled by the gradual incorporation of computational devices into clinical workflows. These tools enable assessments encompassing various aspects, including psychological testing and psychometry [4]. Furthermore, the onset of the COVID-19 pandemic has further supported the adoption of digital tools for telehealth support, particularly within the mental health sector [5]. Digitization also includes aspects that cut across diagnoses, such as socio-anamnestic information relevant to neurodevelopmental conditions [6].

Over the past 2 decades, there has been a significant rise in the application of advanced classification methods, such as supervised machine learning (ML), to enhance diagnostic research in the behavioral sciences [1,3,7-16]. Most of these studies have applied ML-based models to different types of data (eg, home videos and child or adult diagnostic testing), reaching excellent classification accuracies [12-17]. Supervised ML involves the development of algorithms that acquire knowledge from previous experiences to simulate human cognitive processes. ML techniques have been also used to analyze data collected through digital platforms.

In relation to these advancements, recent work from our group has effectively identified children who received a clinical diagnosis of attention-deficit/hyperactivity disorder (ADHD) with an accuracy of up to 82%, using a simple supervised ML approach with decision trees (DT), based on parent- and teacher-reported child behavior data submitted through an internet-based system [3]. Ben-Sasson and Yom-Tov [18] also adopted DT to address a possible early identification of autism spectrum disorder (ASD) through internet-based queries posed by parents suspecting that their children could have that diagnosis; the classifier achieved an area under the curve of 0.82, indicating good predictive accuracy for identifying ASD risk based on parental narratives. Duda et al [19] recruited through crowdsourcing a large sample of parents of children with only ASD or only ADHD to test whether a supervised ML algorithm could differentiate the 2 diagnoses. Using Social Responsiveness Scale items, the linear discriminant analysis and elastic net classifiers achieved an area under the curve of 0.81.

Nevertheless, previous research conducted on adults with ADHD identified accurate classification models based on questionnaire scores. Trognon and Richard [10] developed a psychometric screening scale for the identification of adult ADHD based on *DSM-5* (*Diagnostic and Statistical Manual of Mental Disorders* [Fifth Edition]) diagnostic criteria. They tested an XGBoost classifier to obtain a predictive model for subjects with ADHD compared with controls. The questionnaire scores–based classifier reached an accuracy of 0.98, based on a training set of 154 subjects. Finally, Christiansen et al [11] tested supervised models on the Conners’ Adult ADHD Rating Scale for differentiating between participants with ADHD, obesity, problematic gambling, and a control group. The models reached a predictive accuracy of 0.82 (support vector machine), 0.80 (LightGBM), and 0.79 (logistic regression [LR]).

When testing ML classification models, a relevant focus should be placed on interpretability and accuracy [17]. The first deals with the need for researchers and clinicians to fully understand the relationship between inputs and outputs, which provides an automated decision-making process performed by an ML model. In this context, a model is interpretable when it provides all the critical information about between-variable relationships gained through the learning process. Conversely, accuracy describes how well the ML model performs in providing correct class membership predictions, and it increases with model complexity (hence, with less interpretability) [17]. One or the other must be favored when setting a trade-off between classification accuracy and model interpretability [17]. Traditionally, psychology has primarily focused on explanatory (interpretable) modeling, seeking to understand the causal underpinnings of behavior. However, this emphasis on explanation has often led to models that lack meaningful predictive capacity, raising questions about the robustness and generalizability of psychological research [20].

Within this framework, traditional statistical models like LR have been widely used for clinical classification purposes (ie, for predicting the probability that an observation belongs to 1 of 2 possible classes) because of their open interpretability [17]. Nonetheless, the adoption of less transparent ML methods, such as DT and random forests (RF), has garnered significant attention due to their ability to capture more complex patterns within data. DT and RF often outperform LR, especially when dealing with categorical predictors [18]. Moreover, recent technological advancements enable the enhancement of interpretability in “black box” models using the Shapley additive explanations (SHAP) framework [12]. The SHAP analysis evaluates the influence of classification features in augmenting the likelihood of accurate predictions, allowing researchers to gain more insight into ranking factors that make a diagnosis probable [12].

Given these premises, the hypothesis examined in this study is whether an innovative computational psychometrics framework could exploit the potential of the ML approach to digitally collect data to support the clinical assessment of neurodevelopmental conditions such as ADHD and ASD. To this end, we developed classification ML models to identify either children with ADHD or with ASD using parent-reported socio-anamnestic questionnaires collected through the first Italian internet-based comprehensive screening tool for neurodevelopmental disorders and emotional and behavioral problems, the Medea Information and Clinical Assessment On-Line (MedicalBIT) [6].

## Methods

### Recruitment

**Figure 1:**
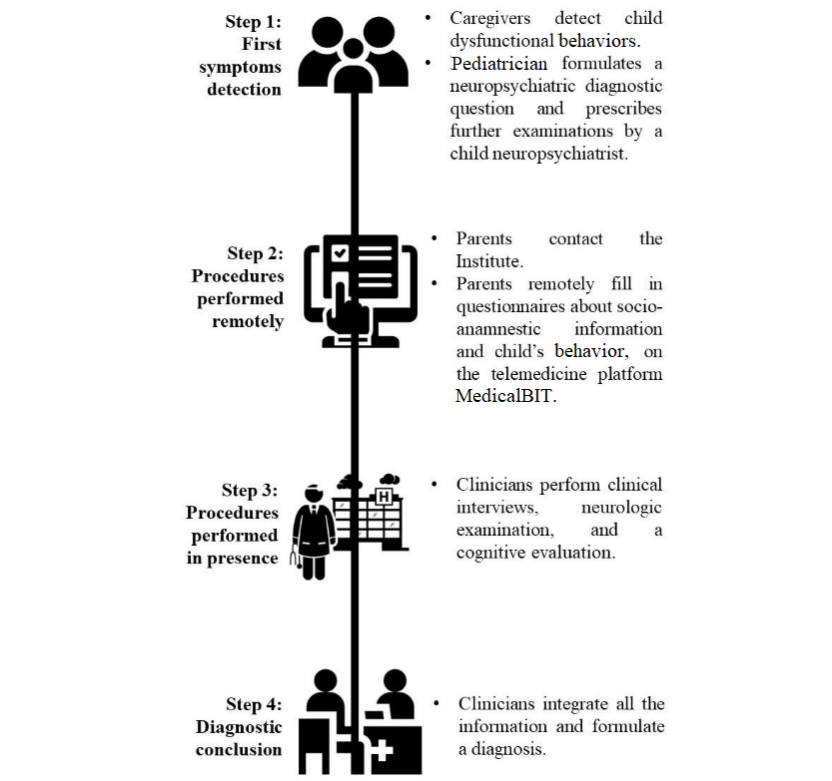
Diagnostic process scheme: the 4 steps from the detection of the first symptoms to the diagnostic conclusion.

In this retrospective, single-center observational study, we considered socio-anamnestic data from a sample of children and adolescents referred for suspected neurodevelopmental conditions at the Scientific Institute “IRCCS Eugenio Medea”—Associazione La Nostra Famiglia in Bosisio Parini (Lecco, Italy)—between October 2018 and May 2022. A workflow of the diagnostic procedure is shown in Figure 1.

### Sample Description

**Figure 2:**
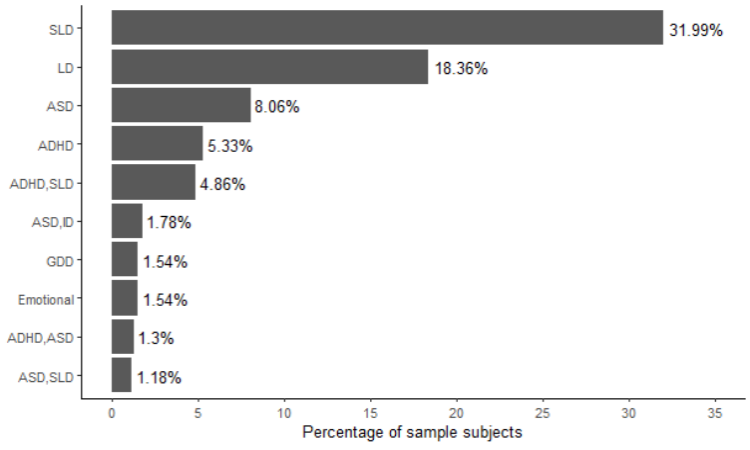
Most frequent configurations of diagnoses across the sample. The figure depicts the configuration of diagnostic categories and comorbidities presented by at least 1% of the participants. ADHD: attention-deficit/hyperactivity disorder; ASD: autism spectrum disorder; GDD: global developmental delay; ID: intellectual disability; LD: language disorder; SLD: specific learning disorder.

Participants included 1688 children and adolescents (n=591, 35% females) living in Northern Italy, aged 2 to 16 years (mean 8, SD 3 years), and who were referred for suspected neurodevelopmental and psychopathological diagnosis. All participant-related data were obtained remotely by parents using the MedicalBIT platform, except for the attending clinician’s categorical diagnosis input into the platform. At our Institute, participants underwent a complete neuropsychiatric evaluation and received one or more clinical diagnoses following the *DSM-5* criteria [19]. In some cases, symptom presentation fell below the diagnostic threshold, and the children did not receive a categorical diagnosis. Possible diagnoses comprised ADHD, ASD, anxiety disorders, communication disorders, developmental co-ordination disorders, emotional and behavioral disorders, intellectual disability, mood disorders, obsessive-compulsive disorder, sleep disorders, and specific learning disorders. Each diagnostic label could represent a single condition or a comorbid presentation (Figure 2). The prevalence of ADHD and ASD diagnoses, regardless of single or comorbid presentations, was equal, accounting for 16% (n=270/1688) of the sample each.

### Independent Variables

The independent variables were derived from a socio-anamnestic questionnaire tapping clinical, biological, environmental, and developmental information. These variables were selected from standard clinical practice and were completed by the participants’ parents before accessing the Institute using the MedicalBIT internet-based platform [6]. It is important to note that the questionnaire items were coded such that higher values corresponded to higher risk levels. Consequently, the item values were aggregated to obtain variables that quantified risk levels in positive familiarity, pre- and perinatal risk, developmental concerns, early health problems, and stressful life events (Table 1).

**Table 1:** List of independent and dependent variables. The 2 dependent variables were considered in separate statistical models.

Area	Variable name	Variable type (values or range)
Sociodemographic	Sex	Dichotomous (male; female)
Positive familiarity	Neurologic or psychopathologic anamnesis in the family Presence of family late talkers	Dichotomous (1=presence; 0=absence)
Pre- and perinatal risk	Preterm birth Pregnancy problems Fertility treatment Smoke during pregnancy: mother Smoke during pregnancy: father Medications during the pregnancy Risk of abortion Birth problems Problems after birth	Dichotomous (1=presence; 0=absence)
	Birth type	Categorical (1=natural; 2=Cesarian; 3=induced; 4=problematic)
	Birth weight	Categorical (0=normal; 1=moderately high or low; 2=extremely low)
	APGAR^a^ at 1 minute APGAR at 5 minutes	Categorical (1=9 or 10; 2=7 or 8; 3=5 or 6; 4=1 to 4)
	Breastfeeding	Dichotomous (0=breastfeeding; 1=formula)
Developmental concerns	Eating habits	Dichotomous (0=normal; 1=selective eating)
	Sleeping problems	Dichotomous (0=no; 1=yes)
	Age of crawling	Categorical (1=6-9 months; 2=9-12 months, 3=>1 year; 4=never)
	Walking	Categorical (1= <12 months; 2=12-18 months, 3=18-24 months; 4= >2 years)
	First words at 18 months Word combinations at 36 months	Dichotomous (0=yes; 1=no)
Early health problems	Any surgery Any audiometry screening Side effects from vaccines	Dichotomous (0=no; 1=yes)
	Severe early health problems	Categorical (0=no; 1=rarely; 2=sometimes; 3=often)
	Early hospitalization	Categorical (0=never; 1=1-2 days, 2=3-4 days, 3=1 week or more)
	Number of early ear infections	Categorical (1=0-2; 2=3-5; 3=6-8; 4=9 or more)
Stressful life events	Any new family member House moving Hospitalization of any family member Death of any family member A parent lost work Severe financial problems Parents separation	Dichotomous (0=no; 1=yes)
Diagnosis	ADHD^b^ diagnosis ASD^c^ diagnosis	Dichotomous (1=presence of diagnosis; 0=absence of diagnosis)

^a^APGAR: appearance, pulse, grimace, activity, and respiration.

^b^ADHD: attention-deficit/hyperactivity disorder.

^c^ASD: autism spectrum disorder.

### Outcome Variables

Clinicians’ diagnostic conclusion regarding the presence or absence of ADHD or ASD was considered as a dichotomous outcome in separate classification models, that are (1) the “ADHD” group comprised participants with a clinical diagnosis of ADHD and possible comorbid conditions; the “non-ADHD” group comprised participants without a clinical diagnosis of ADHD, that is, subjects who received other neuropsychiatric diagnoses or no categorical diagnosis, and (2) the “ASD” group comprised participants with a clinical diagnosis of ASD and possible comorbid conditions; the “non-ASD” group comprised participants without a clinical diagnosis of ASD, that is, subjects who received other neuropsychiatric diagnoses or no categorical diagnosis.

### Statistical Analysis

#### Preliminary Data Handling

Data handling and statistical analyses were done through R software (version 4.1.2; R Core Team) [21]. Missing data were imputed using the 10 nearest neighbors averaging [22].

#### Classification Models

Separate classification models were obtained, addressing the clinical and research questions, that are (1) “should a new help-seeking child be diagnosed with ADHD, considering the parent-reported socio-anamnestic information?” and (2) “should a new help-seeking child be diagnosed with ASD, considering the parent-reported socio-anamnestic information?”

#### Random Forest Models

RF models were performed through the “randomForest” toolbox [23], as previously done [3]. RF is an ensemble learning technique that generates many DTs and aggregates the results. To prevent overfitting, 2 layers of randomness are added in the procedure through bagging: (1) a bootstrap sample of the data set is considered in each tree (the data that are not considered in the bootstrap sample are called out of bag [OOB]); (2) a subset of mtry-independent variables are selected at each tree node. New data categories are predicted by aggregating all predictions performed by the trees, that is, choosing the majority of the voted categories [23]. In the tuning phase of the model selection, a leave-one-out cross-validation (LOOCV) approach was applied [24]. Finally, a SHAP analysis was performed to gain insights into the interpretability of the model [25]. SHAP values are computed by comparing the model’s predictions with and without a particular feature, and this process is repeated iteratively for each feature and sample in the data set. The magnitude of these values reflects the strength of the effect [25].

#### Decision Trees

After conducting RF analyses, DT models were computed. The DT, characterized by a flowchart-like structure, is constructed by considering the entire data set positioned at the top of a “root” node. At each decision point, observations meeting the specified splitting condition are allocated to the left branch, while those not meeting the condition are directed to the right branch [26]. Information gain is a node impurity measure for selecting attributes and dividing each node, continuing until the terminal node, referred to as the “leaf,” is reached [26]. Finally, the algorithm assigns the most frequently observed class in each leaf as the classification prediction [26].

#### Logistic Regression Models

LR models were used in addition to the DT and RF models. LR is a traditional statistical method widely used for binary classification tasks. It models the probability of a binary outcome (presence or absence of the considered diagnosis) based on one or more predictor variables. In our study, LR was applied using the “glm” function in R.

#### Testing the Classification Accuracy

##### Fixed Training and Test Set

To test the classification accuracy of the previously described models, we used 70% of the whole data set as a training set and the remaining 30% as a test set—the 2 subsamples did not present overlapping subjects. The classification performances of the selected models were evaluated considering the following information on the test set:

1. Classification accuracy: percentage of correctly performed classification concerning the total number of instances: 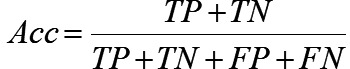;
2. NIR: the no information rate (NIR) represents the largest proportion of the observed classes, indicating the accuracy achievable by always predicting the majority class label.
3. The *P* value of Accuracy>NIR: a hypothesis test result to evaluate whether the classification accuracy performed by the algorithm is greater than the rate of the largest class (NIR).
4. Specificity: percentage of correctly performed negative classification (non-ADHD or non-ASD) concerning the number of subjects without the actual diagnosis:
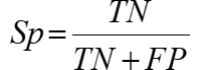
5. Sensitivity: percentage of correctly performed positive classification (ADHD or ASD) for the number of subjects with the actual diagnosis:
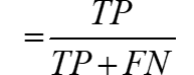
.

##### Five-Fold Cross-Validation

An additional cross-validation step was performed to test the results’ robustness. The whole data set was randomly split into 5 folds, and the 3 classification models were performed on each independent fold. The classification performances were calculated on each test set, and the mean performance values were estimated.

### Ethical Considerations

The study was approved by the Institute’s Ethical Review Board (protocol number 7/23, “Comitato Etico IRCCS E. Medea—Sezione Scientifica Associazione La Nostra Famiglia”). The research was conducted following guidelines and regulations depicted in the Declaration of Helsinki. The study data are deidentified, and no identification of individual participants in any images of the paper is possible. All the participant’s parents or legal guardians were informed of the aim of the study. Each subject was free to participate voluntarily and gave their written informed consent to the minor’s participation. No monetary compensation was provided for participating in the study.

## Results

### Preliminary Data Handling

The maximum percentage of missing data per subject was 36% (3 subjects). Table 2 depicts the sample’s demographic characteristics, considering the total sample and stratification by ADHD and ASD diagnosis.

**Table 2:** Descriptive statistics of demographic variables, considering the whole sample, and stratified by ADHD^a^ and ASD^b^ diagnosis.

Variable		Total sample (N=1688)	ADHD stratification			ASD stratification	
			ADHD (n=269)	Non-ADHD (n=1419)	ASD (n=270)		Non-ASD (n=1418)
Age (years), median (SD)		8 (3)	9 (3)	8 (3)	6 (4)		9 (3)
**Sex, n (%)**							
	Male	1097 (65)	215 (80)	894 (63)	227 (84)		879 (62)
	Female	591 (35)	54 (20)	525 (37)	43 (16)		539 (38)

^a^ADHD: attention-deficit/hyperactivity disorder.

^b^ASD: autism spectrum disorders.

#### Random Forest

**Figure 3:**
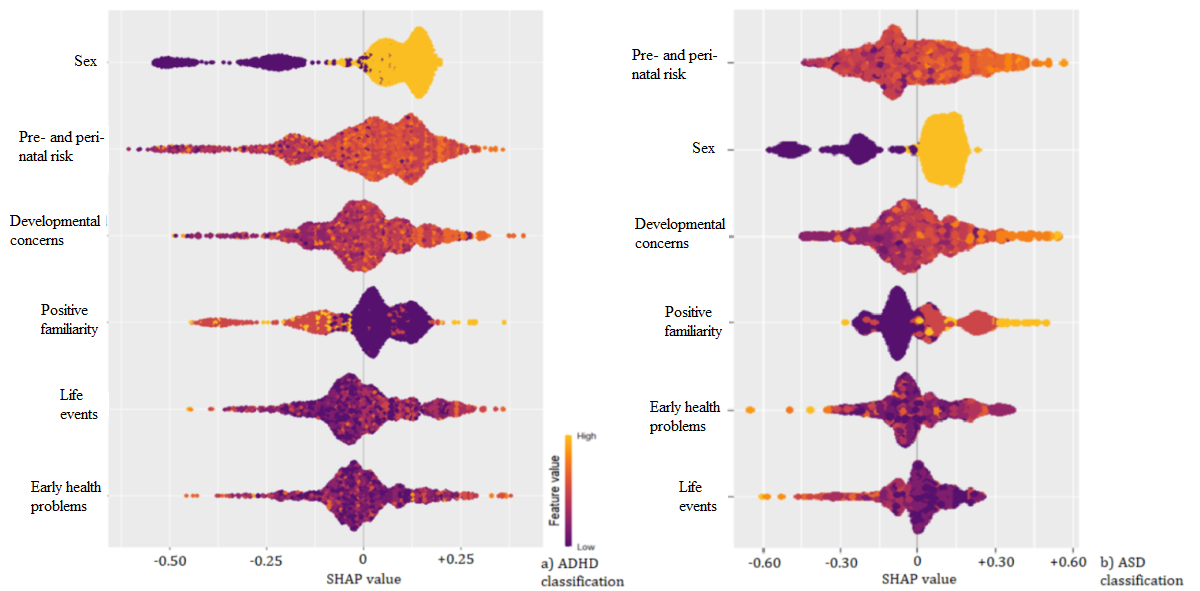
SHAP values for (a) ADHD classification through RF and (b) ASD RF classification through RF. SHAP values are computed by comparing the model’s predictions with and without a particular feature, and this process is repeated iteratively for each feature and sample in the data set. The magnitude of these values reflects the strength of the effect. SHAP: Shapley additive explanations.

Table 3 shows the RF classification models’ performances. Figure 3 shows the SHAP values (ie, the most important independent variables identified by the RF in accurately classifying the diagnoses).

**Table 3:** Performances of the random forest models for classifying attention-deficit/hyperactivity disorder or autism spectrum disorders diagnoses.

Classification model	Performance on the fixed training and test set	SHAP^a^ values, mean (SD)	Average performance on the 5-fold cross-validation sets (SD)
ADHD^b^ vs non-ADHD	Accuracy: 84% (95% CI 82-85) NIR^c^: 50% *P*<.001 Sensitivity: 93% Specificity: 75%	Sex: 0.07 (0.02) Pre- and perinatal risk: 0.03 (0.02) Developmental concerns: –0.02 (0.02) Positive familiarity: 0.02 (0.02) Stressful life events: –0.01 (0.02) Early health problems: –0.01 (0.02)	Accuracy: 68% (2.5) Sensitivity: 73% (2.5) Specificity: 63% (2.5)
ASD^d^ vs non-ASD	Accuracy: 86% (95% CI 84%-87%) NIR: 50% *P*<.001 Sensitivity: 95% Specificity: 77%	Pre- and perinatal risk: 0.08 (0.03) Sex: 0.05 (0.02) Developmental concerns: –0.04 (0.02) Positive familiarity: 0.03 (0.02) Early health problems: –0.02 (0.02) Stressful life events: –0.01 (0.02)	Accuracy: 69% (2.2) Sensitivity: 70% (2.2) Specificity: 68% (2.2)

^a^SHAP: Shapley additive explanations.

^b^ADHD: attention-deficit/hyperactivity disorder.

^c^NIR: no information rate.

^d^ASD: autism spectrum disorders.

#### Decision Tree

Table 4 shows the DT model results and performances on the test sets.

**Table 4:** Performances of the decision tree models in the test set for classifying attention-deficit/hyperactivity disorder or autism spectrum disorders diagnoses.

Classification model	Performance on the fixed training and test set	Attribute importance to the training set, mean (SD)	Average performance on the 5-fold cross-validation sets (SD)
ADHD^a^ vs non-ADHD	Accuracy: 74% (95% CI 71-77) NIR^b^: 50% *P*<.001 Sensitivity: 82% Specificity: 65%	Sex: 0.019 (0.10) Early health problems: 0.008 (0.10) Stressful life events: 0.007 (0.10) Pre- and perinatal risk: 0.004 (0.10) Developmental concerns: 0.001 (0.10) Positive familiarity: 0.001 (0.10)	Accuracy: 59% (3.1) Sensitivity: 57% (3.1) Specificity: 61% (3.1)
ASD^c^ vs non-ASD	Accuracy: 79% (95% CI 77-82) NIR: 50% *P*<.001 Sensitivity: 88% Specificity: 71%	Sex: 0.036 (0.10) Pre- and perinatal risk: 0.016 (0.10) Positive familiarity: 0.013 (0.10) Early health problems: 0.010 (0.10) Developmental concerns: 0.001 (0.10) Stressful life events: 0.001 (0.10)	Accuracy: 64% (3.4) Sensitivity: 67% (3.4) Specificity: 61% (3.4)

^a^ADHD: attention-deficit/hyperactivity disorder.

^b^NIR: no information rate.

^c^ASD: autism spectrum disorders.

#### Logistic Regression

Table 5 shows the LR model results and performances.

**Table 5:** Performances of the logistic regression models for classifying attention-deficit/hyperactivity disorder or autism spectrum disorders diagnoses.

Classification model	Performance on the fixed training and test set	OR^a^ coefficients in the training set (*P*)	Average performance on the 5-fold cross-validation sets (SD)
ADHD^b^ vs non-ADHD	Accuracy: 61% (95% CI 57-64) NIR^c^: 50% *P*<.001 Sensitivity: 62% Specificity: 59%	Sex: 2.14 (*P*<.001) Stressful life events: 1.19 (*P*<.001) Early health problems: 1.09 (*P*=.003) Pre- and perinatal risk: 1.08 (*P*<.001) Developmental concerns: 1.08 (*P*=.016) Positive familiarity: 1.03 (*P*=.676)	Accuracy: 57% (1.9) Sensitivity: 61% (1.9) Specificity: 53% (1.9)
ASD^d^ vs non-ASD	Accuracy: 63% (95% CI 60-67) NIR: 50% *P*<.001 Sensitivity: 68% Specificity: 59%	Sex: 3.88 (*P*<.001) Positive familiarity: 1.93 (*P*<.001) Developmental concerns: 1.17 (*P*<.001) Pre- and perinatal risk: 1.11 (*P*<.001) Early health problems: 1.05 (*P*=.134) Stressful life events: 0.87 (*P*<.001)	Accuracy: 62% (2.1) Sensitivity: 67% (2.1) Specificity: 58% (2.1)

^a^OR: odds ratio.

^b^ADHD: attention-deficit/hyperactivity disorder.

^c^NIR: no information rate.

^d^ASD: autism spectrum disorders.

## Discussion

### Principal Findings

The primary objective of our study was to develop accurate classification models for the diagnosis of ADHD and ASD within a sample referred for clinical evaluation. To this end, we used an ML approach to analyze internet-based parent-reported socio-anamnestic questionnaires.

Our ML models reached overall reasonable classifications in the test sets for both ADHD and ASD. The RF models exhibited classification accuracies of 84% for ADHD and 86% for ASD, respectively, with high sensitivities (93% for ADHD and 95% for ASD). On the other hand, the DT and LR models reached lower accuracy rates, with 74% and 61% accuracy for ADHD and 79% and 63% for ASD, respectively. The DT and LR models also demonstrated lower sensitivities (82% and 62% for ADHD and 88% and 68% for ASD).

In the 5-fold experiment, all models showed a decline in predictive accuracy, as could be expected due to smaller sample sizes. Nevertheless, the RF model continued to exhibit greater accuracy than other models. Concerning the different levels of accuracy reached by our 3 ML models, it is crucial to acknowledge both the advantages and disadvantages of RF, DT, and LR. One of the distinctive features of RF models is that they can effectively capture complex relationships within the data that may elude human interpretation [17]. For this reason, RF models can occasionally be considered difficult to interpret. This characteristic needs adequate consideration in the clinical context because the primary aim is to provide clinicians with an accurate “first glance” tool that supports them in forming initial diagnostic impressions.

Notwithstanding their eventual interpretability, RF models are remarkably effective in distinguishing different classes, thus representing an asset in psychopathology diagnosis. Conversely, as mentioned above, the DT and LR models are also readily interpretable for clinicians less familiar with ML techniques [17]. Therefore, the choice of approach depends on the decisional context and the desired degree of interpretability. In this study, we preferred greater levels of classification accuracy over the readiness of the classification process. However, a noteworthy option to mitigate the interpretability concern associated with RF models is provided by SHAP analysis. By assigning an important value to each feature in the classification model, SHAP analysis directly compares RF and other models regarding their interpretability.

Although slightly different in the achieved performance, the 3 models identified sex as the strongest predictor for both ADHD (all 3 models) and ASD (DT and LR models). It is well documented that males are more likely to be diagnosed with both ADHD [27] and ASD [28] than females. Interestingly, SHAP analysis indicated a relatively consistent ranking of features for RF models across the 2 clinical diagnoses. After sex, which showed by far the highest discriminative ability among the cases, the presence of pre- and perinatal risk and other developmental concerns featured as influential predictors of both ADHD and ASD classes. Not surprisingly, given the significant heritability of the 2 conditions, having a family member with reported difficulties was also a relevant predictor of the classification.

On the other hand, DT and LR models identified feature rankings that were, except for sex, significantly different for ADHD and ASD classification. This discrepancy could be due to the underlying assumptions of the different ML methodologies. Whereas LR models assume linear relationships between predictors and outcomes, DT and RF models could exploit nonlinear relationships and interactions within the data [18]. Consequently, some degree of variation in predictor ranking is expected, further highlighting the diverse nature of insights gained from different analytical methodologies. Finally, it should be remembered that it is impossible to conclude the causality and direction of the interrelations among predictors in the ML model.

### Comparison With Previous Work

Our RF model’s accuracy was in line with previous ML classification approaches to questionnaire data [10,11,15,16,29] and other data sources [30-37].

Nevertheless, these classification models outperformed recent work from our group, where we identified children with ADHD with an accuracy of up to 82% using a DT-based supervised ML approach [3]. Despite some methodological differences, the higher level of accuracy obtained in the current work underscores the potential of RF models in increasing the precision of computer-aided diagnosis. Altogether, this pattern of findings suggests that the RF model outperformed both the DT and LR models in effectively categorizing neurodevelopmental conditions based on parent-reported socio-anamnestic information, as highlighted by previous studies [27,28,38].

### Implications for Clinical Practice and Future Research

In the domain of child and adolescent neuropsychiatry, the diagnostic process includes an initial stage where anamnestic, sociodemographic, and behavioral data need to be collected. This data gathering can be remotely performed through internet-based parent reports, as evidenced by previous studies [5,6]. With this regard, the MedicalBIT platform currently represents the first Italian internet-based screening instrument for child and adolescent neuropsychiatric conditions [6]. As the data are compiled in databases within MedicalBIT, the exploitation of ML models can prompt the classification of the probable diagnostic risk associated with new subjects seeking assistance. The significant predictive value of the models developed in this study might be valuable to support the clinical practice of diagnosing neurodevelopmental conditions.

### Limitations

Despite the encouraging findings, this study is not free of limitations. First, our ML models exclusively rely on parent-reported data. Existing literature [37] has previously indicated that the reliability of these reports could be negatively influenced by factors such as the possibility of accessing digital tools, intrinsic comprehension difficulties, or general parental educational attainment. Second, our sample exclusively included children and adolescents from a geographically restricted region (Northern Italy). The generalizability of current findings to populations from different areas needs cautious consideration. Third, the relatively low occurrence of ADHD-ASD comorbidity in our cohort prevents us from developing classification models tailored for more nuanced diagnostic presentations, such as either ADHD- or ASD-only versus ADHD-ASD comorbid presentation. Therefore, future extensions of this study should consider including broader cohorts of participants to consider this possibility.

### Conclusions and Future Advancements

Within the rapidly evolving context of “psycho-informatics,” we believe that the current work represents a noteworthy effort in the realm of computational psychometrics [28]. Through an exploration of remotely collected parent-reported socio-anamnestic data, the current research has revealed promising avenues for enhancing the diagnostic process of neurodevelopmental and psychopathological conditions. Integrating digital platforms for data collection and ML could offer clinicians a dynamic tool supporting their diagnostic decisions. Within the health care systems, clinical teams confront a scarcity of personnel, with high emotional and cognitive demands for the actual staff [38]. In this context, this research represents a preliminary effort to mitigate the clinicians’ workload by automating specific tasks (such as data collection and analysis). If proven effective, this approach could leave more time for clinicians to nurture the essential patient-clinician bond, a facet that remains irreplaceable by artificial intelligence technologies.

## Abbreviations

ADHD: attention-deficit/hyperactivity disorder
ASD: autism spectrum disorder
DSM-5: Diagnostic and Statistical Manual of Mental Disorders (Fifth Edition)
DT: decision tree
LOOCV: leave-one-out cross-validation
LR: logistic regression
MedicalBIT: Medea Information and Clinical Assessment On-Line
ML: machine learning
NIR: no information rate
OOB: out of the bag
RF: random forest
SHAP: Shapley additive explanations

## Data Availability

The data sets analyzed during this study are available from the corresponding author on reasonable request.
